# Comprehensive Review of *Perilla frutescens*: Chemical Composition, Pharmacological Mechanisms, and Industrial Applications in Food and Health Products

**DOI:** 10.3390/foods14071252

**Published:** 2025-04-03

**Authors:** Dandan Yi, Zhiyong Wang, Mu Peng

**Affiliations:** College of Biological and Food Engineering, Hubei Minzu University, Enshi 445000, China; gmx15565575786@gmail.com (D.Y.); wangzhiyong@hbmzu.edu.cn (Z.W.)

**Keywords:** *Perilla frutescens*, chemical composition, functional activity, functional foods

## Abstract

*Perilla frutescens* (L.) Britt., a multifunctional herbaceous plant, is widely used in traditional medicine and cuisine due to its rich array of bioactive compounds. To date, many key phytochemicals in *P. frutescens* have been identified, including volatile terpenoids (perillaldehyde, limonene,), flavonoids (luteolin, apigenin), and phenolic acids (rosmarinic acid derivatives), which exhibit significant antioxidant, anti-inflammatory, antiviral, anticancer, antibacterial, and blood sugar-lowering effects. Studies have shown that volatile oils, flavonoids, and phenolic acids in *P. frutescens* exert their effects in various experimental models. In food and industrial applications, *P. frutescens* shows innovative potential in functional foods, natural preservatives, and novel food additives, effectively extending food shelf life and providing antimicrobial protection. Moreover, research on the biology and genetic improvement of *P. frutescens* has provided new approaches to enhance its yield and bioactive content. Finally, this paper also discusses the safety and standardization issues of the plant, providing theoretical support for its widespread application.

## 1. Introduction

**Figure 1:**
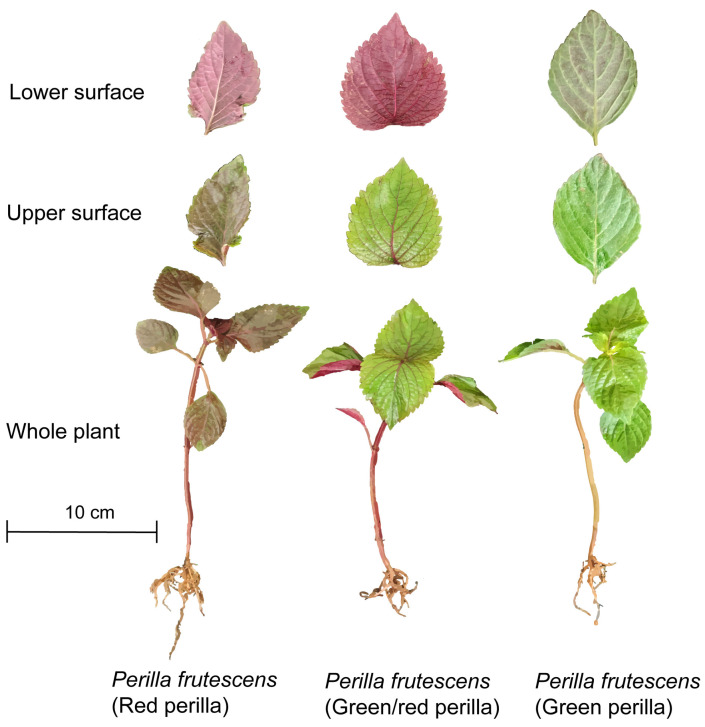
Leaf and whole plant morphology of *Perilla frutescens*. Red (purple) perilla refers to plants with red (purple) leaves. Green perilla has entirely green leaves. Green/red perilla has green leaves with purple leaf backs.

*Perilla frutescens* (L.) Britt. is an annual, erect herbaceous plant of the genus *Perilla* in the Lamiaceae family, widely distributed across Asia. It has been traditionally employed in medicinal and culinary practices for centuries across China, Japan, Korea, and other Asian countries [1]. Leaf color is a critical attribute for consumer preference in *P. frutescens* [2]. The plant species *P. frutescens* exhibits three distinct chemotypes: green, bicolored (green/red), and red (purple), characterized by differential pigmentation patterns during leaf and stem development [3,4]. Red perilla manifests uniform anthocyanin expression across both adaxial (upper) and abaxial (lower) leaf surfaces, presenting as dark erythroid pigmentation, while the bicolored phenotype displays this chromatic characteristic exclusively on the abaxial surface (Figure 1). The purple and green/purple *P. frutescens* leaves are considered medicinal, while the green leaves are considered edible [5]. As both a medicinal plant and an industrial crop, *P. frutescens* is extensively employed in the food, pharmaceutical, and cosmetic industries, largely due to its distinctive aromatic compounds [6].

*P. frutescens* is a versatile medicinal and edible plant with extensive utilization potential, as its leaves, stems, and seeds possess distinct bioactive properties [6]. Tender leaves are eaten fresh in salads, brewed into herbal tea, or added to soups, while the stems and leaves are commonly used for pickling. In traditional Chinese cuisine, *P. frutescens* leaves are frequently paired with seafood to alleviate gastrointestinal discomfort. Notably, *P. frutescens* leaf decoctions are traditionally consumed in summer to reduce heat stress and stimulate appetite [7]. In Japanese and Korean cuisines, fresh *P. frutescens* leaves are commonly used as a raw vegetable or garnish.

Comprehensive phytochemical studies have identified key bioactive constituents of *P. frutescens*, including volatile oils (perillaldehyde, limonene), flavonoids (apigenin, luteolin), phenolic acids (rosmarinic acid, caffeic acid), and triterpenoids. These bioactive compounds contribute to its diverse pharmacological activities, including antimicrobial, antiviral, anticancer, anti-inflammatory, antioxidant, hypoglycemic, and neuroprotective effects [8,9,10,11]. A variety of *P. frutescens*-derived products, including *P. frutescens* seed oil (with an omega-3 content exceeding 60%), functional teas, and alcoholic beverages, have gained prominence in niche markets, benefiting from the plant’s GRAS (Generally Recognized As Safe) status.

This review aims to integrate interdisciplinary research progress on *P. frutescens*, with a focus on its chemical composition, pharmacological mechanisms, applications in the food industry, and advances in synthetic biology. By addressing current research gaps and outlining future industrial development pathways, we propose a comprehensive “Resource Development-Mechanistic Investigation-Technological Transformation-Standard Regulation” framework. This framework offers a structured and scientific roadmap for the comprehensive exploration of *P. frutescens*’s potential, ensuring its safety, regulatory compliance, and long-term sustainability. This proposed framework not only optimizes the multifaceted value of *P. frutescens* but also sets a benchmark for the innovation and advancement of natural food additives. With its implementation, *P. frutescens* is expected to evolve from a traditional herb into a globally competitive health food ingredient, contributing to the green transformation of the food industry. The research direction of *P. frutescens* in the latest five years are shown in Table 1.

## 2. Chemical Composition

### 2.1. Volatile Oils

The essential oils of *P. frutescens*, predominantly composed of monoterpenes and sesquiterpenes, possess distinctive aromatic profiles and significant bioactivities, rendering them highly versatile in various applications [12,13]. Comprehensive phytochemical analyses have identified key constituents such as perillaldehyde, perilla ketone, limonene, shisofuran, farnesenes (Z, E, α), and trans-shisool [14]. GC-MS analysis identified perilla ketone as the dominant volatile constituent, constituting 80.76% of the essential oil profile. Other components included β-caryophyllene (1.65%), linalool (1.15%), caryophyllene oxide (1.12%), and apiol (1.19%) [8]. Among these compounds, perillaldehyde is the most characteristic component of *P. frutescens* leaf essential oil, distinguished by its strong herbal aroma and potent antimicrobial and anti-inflammatory properties [15]. Limonene, another key monoterpene, possesses diverse biological activities, including anticancer, antioxidant, and antiviral effects [16,17]. Additionally, perilla alcohol and β-caryophyllene have been validated for their pharmacological potential in preclinical models [1]. The composition of *P. frutescens* essential oils (PEOs) is subject to various environmental factors, including humidity, soil conditions, temperature, seasonal variations, harvest timing, sowing period, geographical distribution, and phenological stages [18]. Notably, PEOs modulate key targets such as SLC6A4 (serotonin transporter) and SLC6A3 (dopamine transporter), regulating serotonergic and dopaminergic synaptic pathways and thereby alleviating menopause-associated depression [19]. Additionally, PEOs exhibit potent larvicidal activity against *Aedes aegypti mosquitoes*, leading to a 68% reduction in dengue fever transmission rates in endemic regions [20]. PEOs have been formulated into bioinsecticides, exhibiting effectiveness against *Tribolium castaneum* and *Lasioderma serricorne* [21].

**Figure 2:**
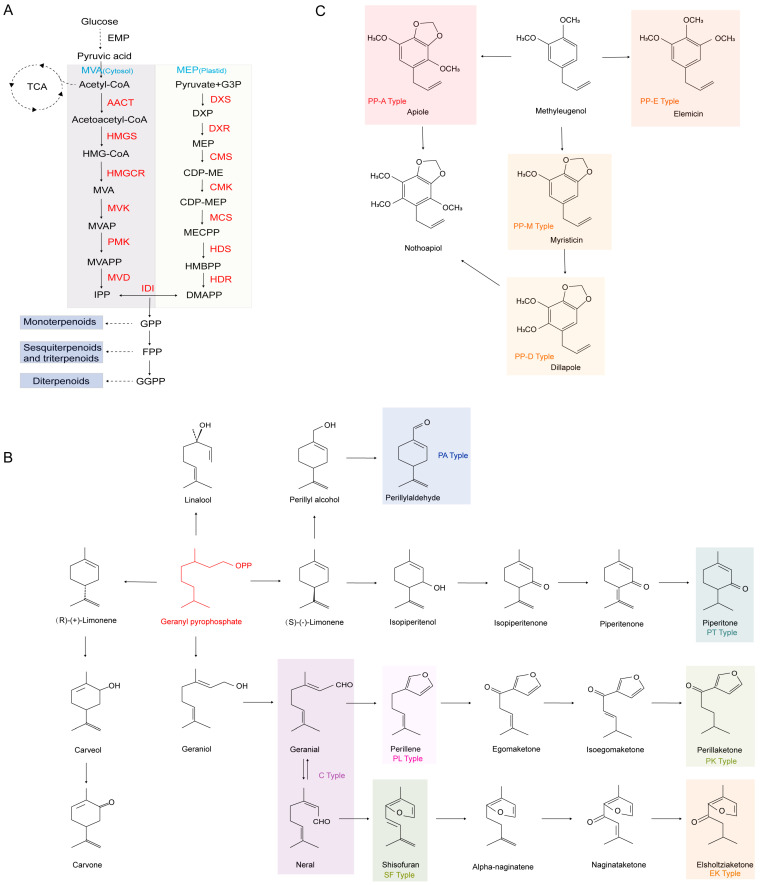
(**A**) *Perilla frutescens* terpene synthesis pathway; (**B**,**C**) *P. frutescens* chemotype. EMP, glycolytic pathway; TCA, tricarboxylic acid cycle; AACT, acetyl-CoA acetyltransferase; HMGS, hydroxymethylglutaryl-CoA; HMGCR, hydroxymethylglutaryl-CoA reductase synthase; MVK, mevalonate kinase; PMK, phosphomevalonate kinase; MVD, mevalonate diphosphate decarboxylase; DXS, 1-deoxy-D-xylulose-5-phosphate synthase; DXR, 1-deoxy-D-xylulose-5-phosphate reductoisomerase; CMS, 2-C-methyl-D-erythritol 4-phosphate cytidylyltransferase; CMK, 4-diphosphocytidyl-2-C-methyl-D-erythritol kinase; MCS, 2-C-methyl-D-erythritol 2,4-cyclodiphosphate synthase; HDS, 4-hydroxy-3-methylbut-2-enyl diphosphate synthase; HDR, 4-hydroxy-3-methylbut-2-enyl diphosphate reductase; IDI, isopentenyl pyrophosphate isomerase; IPP, isopentenyl pyrophosphate; DMAPP, dimethylallyl pyrophosphate; GPP, geranyl pyrophosphate; FPP, farnesyl pyrophosphate; GGPP, geranylgeranyl pyrophosphate.

The biosynthesis of PEOs is predominantly driven by terpenoid metabolism, wherein monoterpenoids (such as perillaldehyde and limonene) are synthesized through the coordinated action of the mevalonate (MVA) and methylerythritol phosphate (MEP) pathways (Figure 2A). Typically, the MVA pathway operates in the cytoplasm of eukaryotic cells, whereas the MEP pathway functions within the plastids of plants and prokaryotes. In this process, glucose enters the cell and undergoes glycolysis in the cytoplasm, yielding pyruvate, which is subsequently converted into acetyl-CoA within the mitochondria. Acetyl-CoA then enters the MVA pathway to generate isopentenyl pyrophosphate (IPP), while its isomer, dimethylallyl pyrophosphate (DMAPP), is produced via the MEP pathway. These two intermediates serve as precursors for the synthesis of geranyl pyrophosphate (GPP), the fundamental building block for monoterpenoid biosynthesis. Under the catalysis of specific enzymes, GPP gives rise to various constituents of PEOs, leading to the classification of different chemical types (Figure 2B,C).

### 2.2. Flavonoids

*P. frutescens* is acknowledged as a valuable source of structurally diverse flavonoids, which have been extensively studied for their pharmacological properties. Wang et al. [22] identified three primary flavonoid aglycones (luteolin, apigenin, and 5,7,2′,5′-tetrahydroxyflavone), five flavonoid glycosides (luteolin-7-*O*-diglucuronide, apigenin-7-*O*-diglucuronide, luteolin-7-*O*-glucuronide, apigenin-7-*O*-glucuronide, and quercetin-3-*O*-β-D-glucuronide), and two anthocyanins (shisonin and malonylshisonin) in *P. frutescens*. Among these, luteolin (3′,4′,5,7-tetrahydroxyflavone) and apigenin (4′,5,7-trihydroxyflavone) are the principal bioactive flavonoids, demonstrating potent anticancer and anti-inflammatory activities [23,24,25]. Luteolin and apigenin are structurally homologous flavonoids, both possessing a common flavone backbone, while the presence of a 3′-hydroxyl group in luteolin’s B-ring confers greater lipophilicity relative to apigenin [26]. The flavonoid content in leaves, stems, and seeds varies considerably, and fluctuations in these levels can influence the overall antioxidant potential of *P. frutescens* [27]. *P. frutescens* leaves contain significantly higher levels of flavonoids compared to stems, with the flavonoid content in leaves exceeding twice that in stems. Furthermore, the total flavonoid content in leaves is approximately five times greater than in stems.

### 2.3. Phenolic Acids

*P. frutescens* is rich in diverse phenolic acids, including rosmarinic acid, caffeic acid, and their derivatives, such as 3′-dehydroxyrosmarinic acid-3-*O*-glucoside and rosmarinic acid-3-*O*-glucoside. Additional identified compounds include methyl rosmarinate, caffeic acid-3-*O*-glucoside, various caffeic acid esters, vanillic acid, protocatechuic acid, and chlorogenic acid [28,29]. Among these, *P. frutescens* leaves exhibit the highest concentration of phenolic acids, significantly surpassing those found in stems and seeds. *P. frutescens* floral tissues harvested at 20 days post anthesis demonstrate maximal phenolic compound yields, with rosmarinic acid constituting the predominant phytochemical component during this developmental phase [30]. Phytochemical profiling revealed significant interspecific and organ-specific variations, with rosmarinic acid identified as the predominant phenolic constituent in seed extracts across analyzed taxa (4606.4 μg/g). Strikingly, Perilla frutescens seeds exhibited exceptional phenolic accumulation, achieving the maximal total phenolic content of 4856.8 μg/g DW. Compared to other plant parts, such as the main stem, lateral stem, and petiole, both perilla leaves and roots also exhibit high phenolic content, ranging from 272.4 to 886.9 μg/g and 354.7 to 993.3 μg/g, respectively [10].

**Figure 3:**
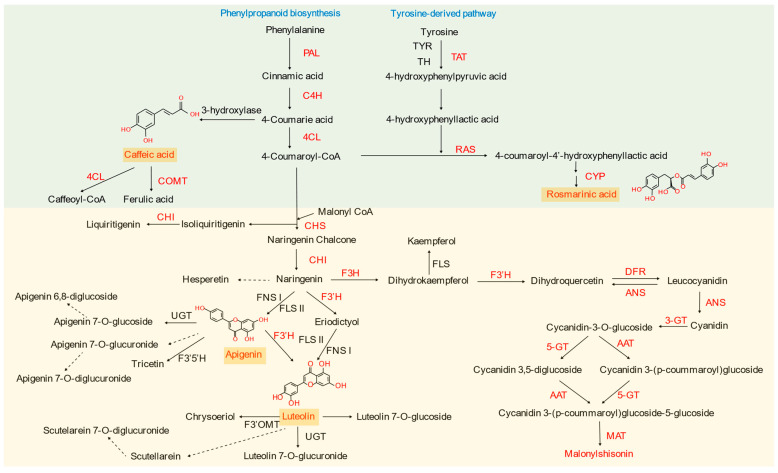
Synthesis pathway of Perilla frutescens flavonoids and phenolic acids. PAL, phenylalanine ammonia lyase; C4H, cinnamate 4-hydroxylase; 4CL, 4-coumarate-CoA ligase; COMT, caffeic acid 3-*O*-methyltransferases; CHS, chalcone synthase; CHI, chalcone isomerase; F3H, flavanone 3-hydroxylase; F3′H, flavonoid 3′-hydroxylase; FNSI, flavone synthasel, I; FLSII, flavone synthase II; UGT, UDP-glycosyltransferase; F3′5′H, flavonoid 3′,5′-Hydroxylase; TAT, tyrosine aminotransferase; RAS, rosmarinate synthase; CYP, cytochrome P450; DFR, dihydroflavonol reductase; ANS, anthocyanin synthase; 3-GT, flavonoid 3-*O*-glucosyltransferase; AAT, anthocyanin acyltransferase; 5-GT, anthocyanin 5-*O*-glucosyltransferase; MAT, Malonyl-CoA: anthocyanin malonyltransferase.

Studies have shown that *P. frutescens* polyphenol extracts enhance the freshness, herbal aroma, and overall sensory acceptability of grass carp (*Ctenopharyngodon idella*) by mitigating fishy odors resulting from lipid oxidation during cooking [31]. Both caffeic acid and rosmarinic acid enhance the hepatic synthesis of endogenous antioxidant enzymes and glutathione (GSH); however, rosmarinic acid demonstrates a superior ability to scavenge DPPH free radicals compared to caffeic acid [32,33]. Proton ionic liquids, in combination with microwave-assisted techniques, have been employed to extract rosmarinic acid with a purity reaching 90.4% [34]. Moreover, rosmarinic acid has been found to inhibit inflammatory responses and disrupt the metabolic and glycolytic pathways of Trichophyton mentagrophytes [35]. The synthesis pathway of *P. frutescens* flavonoids and phenolic acids is shown in Figure 3.

### 2.4. Anthocyanins

Anthocyanins exhibit a wide range of biological activities in traditional Chinese medicine, including antioxidative, antihypertensive, anti-inflammatory, anti-atherosclerotic, and anticancer effects. In *P. frutescens*, the anthocyanin profile predominantly consists of cyanidin-type derivatives, with malonylshisonin being the most abundant component followed by shisonin. The pigmentation of *P. frutescens* leaves is primarily attributed to anthocyanins; however, these compounds are highly susceptible to degradation under high pH, light exposure, heat, and ionizing substances [36]. Light conditions play a crucial role in anthocyanin biosynthesis, with red and blue light treatments enhancing anthocyanin accumulation by 4.3-fold compared to green light exposure [37]. In the red (purple) perilla, anthocyanins are responsible for the deep red and purple hues of the leaves, while in green perilla, anthocyanins are either absent or present in very low concentrations, resulting in entirely green leaves. The green/red perilla variety exhibits a unique combination of green leaves with purple undersides, where anthocyanins accumulate predominantly on the leaf backs. These differences in anthocyanin content and distribution are crucial for distinguishing and classifying the various *P. frutescens* varieties. Moreover, anthocyanins have been reported to regulate glucose metabolism, enhance insulin sensitivity, protect against ultraviolet-induced damage, and facilitate collagen synthesis [38].

### 2.5. Fatty Acids

*P. frutescens* seeds exhibit high concentrations of fatty acids, predominantly composed of palmitic acid (PA, C16:0), stearic acid (SA, C18:0), oleic acid (OA, C18:1), linoleic acid (LA, C18:2), and α-linolenic acid (ALA, C18:3). Notably, the seed oil of *P. frutescens* serves as an exceptional botanical source of polyunsaturated fatty acids (PUFA), distinguished by its uniquely high α-linolenic acid content. Accounting for 54–64% of total fatty acids, the ALA proportion in *P. frutescens* seed oil significantly surpasses that of most other plant-derived oils, establishing it as a preeminent natural reservoir of omega-3 fatty acids [6]. Fatty acid desaturase 3 (FAD3) serves as a critical enzymatic mediator in PUFA biosynthesis, specifically catalyzing the desaturation of linoleic acid into α-linolenic acid. Notably, genetic variations in FAD3, particularly single nucleotide polymorphisms (SNPs), have been strongly associated with quantitative alterations in fatty acid profiles, demonstrating significant correlations with LA and ALA content modulation across diverse biological systems [39]. Currently, *P. frutescens* seed oil has gained prominence as a natural functional edible oil, with its development and utilization expanding rapidly in both nutraceutical and culinary applications.

### 2.6. Polysaccharides

A novel polysaccharide (PFP) isolated from Perilla frutescens exhibits a unique compositional profile, comprising 89.73% neutral sugars, 8.93% uronic acid, and 1.56% protein, with a molecular weight of 1.18 × 10^6^ Da. Integrated spectroscopic (FT-IR, NMR) and ion chromatographic analyses confirmed the pyranose configuration of PFP, revealing a monosaccharide composition of rhamnose, arabinose, galactose, glucose, xylose, and galacturonic acid in molar ratios of 0.13:0.55:1.40:1.00:0.13:0.22. Structural modeling suggests a backbone comprising α-L-Araf-(1→, →6)-β-D-Galp-(1→, →4)-α-D-Glcp-(1→, →1,4)-β-D-Xylp-(1→, →4)-α-GalpA-(1→, →2,4)-α-L-Rhap-(1→. Functionally, PFP demonstrates dual therapeutic potential by significantly inhibiting the proliferation of H22 hepatoma cells while concurrently mitigating chemotherapy-induced immunosuppressive effects on immune organs, highlighting its promise as a multifunctional adjuvant in oncological interventions [40].

## 3. Functional Activity

### 3.1. Antioxidant

The antioxidant capacity of *P. frutescens* is primarily assessed using in vitro assays that measure its free radical scavenging ability. A comparative analysis of 12 *Perilla* cultivars demonstrated substantial variability in their antioxidant activity [41]. The potent antioxidant properties of *P. frutescens* are attributed to its abundance of flavonoids, phenolic acids, and essential oils. Moreover, metabolite composition is a key determinant of *P. frutescens* leaf antioxidant activity, with higher metabolite levels correlating with enhanced antioxidant capacity [42].

Among the various *P. frutescens* varieties, purple-leaved *P. frutescens* demonstrates the highest antioxidant activity, primarily attributed to its elevated rosmarinic acid content (314.3 mg/g) [43]. Cold-pressed *P. frutescens* oil (CPPO) demonstrates significant anti-photoaging efficacy, effectively attenuating ultraviolet-induced wrinkle formation and melanin deposition in hairless murine models. Mechanistic studies revealed its potent antioxidant capacity through dual modulation of oxidative stress markers: suppressing reactive oxygen species (ROS) generation while downregulating superoxide dismutase (SOD) activity in normal human dermal fibroblasts (NHDFs). These dose-dependent effects (0.625–0.25%) confirm CPPO’s multimodal antioxidant-photo-protective properties [44].

Perillaldehyde, a key constituent of PEO, inhibits ROS production and exerts antioxidant effects by activating the nuclear factor erythroid 2-related factor 2 (NRF2) and heme oxygenase-1 (HO-1) pathways in human keratinocytes [45]. Caffeic acid and rosmarinic acid enhance hepatic antioxidant enzyme activity, thereby exerting protective effects [32,46]. Luteolin has been reported to attenuate ROS generation and promote neuronal survival [47].

### 3.2. Anti-Inflammatory

*P. frutescens* demonstrates strong anti-inflammatory properties against multiple inflammatory disorders, including otitis, hepatitis, colitis, and airway inflammation [48]. The anti-inflammatory potential of *P. frutescens* has been predominantly investigated in animal models. *P. frutescens* extract (containing caffeic acid, rosmarinic acid, luteolin, and apigenin) from the whole plant markedly inhibits key inflammatory transcriptional regulators, such as nuclear factor kappa B (NF-κB) and signal transducer and activator of transcription 3 (STAT3), thereby mitigating colitis symptoms in mice [49]. Additionally, it reduces the phosphorylation of Bruton’s tyrosine kinase (Tyr223 and Tyr174) in fMLF-activated human neutrophils while lowering intracellular Ca^2+^ levels [50].

*P. frutescens* seed oil exhibits inhibitory effects on ethyl phenylpropiolate (EPP)-induced auricular edema in rats, demonstrating anti-inflammatory activity. However, due to the required pharmacokinetic processes including absorption, metabolism, and systemic distribution to target cells, the inhibition rates of *P. frutescens* seed oil reached only 59.3% (0.1 mL/ear) and 65.7% (1 mL/ear) at the 15 min time point [51]. Moreover, perillaldehyde, perilla ketone, isoperilla ketone, ursolic acid, rosmarinic acid, and apigenin have been reported to modulate macrophage inflammatory responses via multiple pathways, with their mechanisms extensively studied [52,53,54]. In an arthritis mouse model, mutant *P. frutescens* leaf extract (SFE-M) significantly reduced the neutrophil-to-lymphocyte ratio (NLR) in whole blood and markedly diminished inflammatory cell infiltration and edema formation [55].

### 3.3. Antiviral

*P. frutescens* harbors a diverse array of antiviral compounds, including caffeic acid, rosmarinic acid, luteolin, ferulic acid, apigenin-7-*O*-diglucuronide, perilla alcohol, menthol, perillaldehyde, baicalin, eugenol, safrole, catechaldehyde, methyl caffeate, and cinnamaldehyde [56]. These compounds exert potent inhibitory effects on the replication and dissemination of various viruses.

*P. frutescens* leaf extract prevents viral entry into host cells by inactivating viral particles, exhibiting an EC50 of 0.12 ± 0.06 mg/mL and a CC50 (drug concentration causing 50% cytotoxicity as determined by the MTT assay) of 4.64 ± 0.16 mg/mL against severe acute respiratory syndrome coronavirus 2 (SARS-CoV-2) [57]. Luteolin regulates the NF-κB/STAT3/ATF6 signaling pathway, effectively suppressing African swine fever virus (ASFV) replication [58]. It also exhibits strong antiviral activity against the largemouth bass virus (LMBV), with a viral replication inhibition rate of 97.27%. Its half-maximal inhibitory concentration (IC50) in epithelioma papulosum cyprini (EPC) cells is 2.77 μM, highlighting its potential in aquatic viral disease treatment [59].

Perilla alcohol and perilla acid do not affect viral genomic replication but act in the later stages of the herpes simplex virus type 1 (HSV-1) lifecycle, inhibiting the release of infectious viral particles from Vero cells [60]. Apigenin does not influence the viral lifecycle (e.g., attachment, entry, or budding) but directly inhibits viral polymerase activity [24]. These findings highlight the potential of *P. frutescens* as a natural plant with antiviral properties and provide important insights for the development of novel antiviral drugs.

### 3.4. Anticancer

The anticancer properties of *P. frutescens* primarily arise from its bioactive constituents, including perillaldehyde and rosmarinic acid, which modulate inflammatory signaling pathways (e.g., NF-κB) and reduce oxidative stress [61]. Cancer metastasis remains a significant challenge in oncology. Studies have demonstrated that *P. frutescens* (stems, leaves, and seeds) extracts (rosmarinic acid, perillaldehyde) suppress adrenaline-induced cancer cell metastasis [62] and exert significant cytotoxic effects on prostate cancer cell lines [63]. Rosmarinic acid exhibits anticancer effects through two primary mechanisms: inhibition of inflammatory responses and scavenging of ROS [64]. The anticancer mechanisms of luteolin, a key flavonoid, have been comprehensively reviewed elsewhere [65].

Notably, nanotechnology-based formulations, such as *P. frutescens* flavonoid-stabilized silver nanoparticles (PFFE-AgNPs) and rosmarinic acid-functionalized silver nanoparticles (PFRAE-AgNPs), exhibit strong anticancer effects against multiple human cancer cell lines, including COLO205 (colon adenocarcinoma), PC-3 (prostate cancer), A549 (lung adenocarcinoma), and SKOV3 (ovarian cancer) [66,67]. Furthermore, recent studies indicate that probiotic strains *P. pentosaceus* (F1), *L. fermentum* (F24), and *P. acidilactici* (F23) enhance the accumulation of phenolic compounds in *P. frutescens*, thereby amplifying its anticancer effects through phytochemical synergy [9].

### 3.5. Antibacterial

*P. frutescens* possesses broad-spectrum antimicrobial properties, effectively inhibiting various bacterial pathogens, including *Escherichia coli*, *Staphylococcus aureus*, *Streptococcus pneumoniae*, *Shigella* spp., and *Salmonella* spp. [68]. Additionally, PEO exhibits antifungal activity against several plant pathogenic fungi, such as *Aspergillus flavus*, *Aspergillus niger*, *Aspergillus oryzae*, *Fusarium solani*, and *Rhizoctonia solani* [69]. Proteomic analysis reveals that *P. frutescens* oil inhibits the growth of *A. flavus* by impairing antioxidant defense mechanisms and disrupting glycolytic pathways. This effect is supported by the marked upregulation of ribosomal proteins (Kri1, Noc4, Rlp24, Ytm1, Mak21, Ssf2, and Tsr3), along with enhanced enzymatic activity linked to RNA transcription and translation in *A. flavus* following *P. frutescens* oil treatment [70].

*P. frutescens* contains bioactive compounds, including rosmarinic acid, caffeic acid, perillaldehyde, luteolin, and other phenolic acids, which demonstrate strong antibacterial and antifungal activities [1,35]. Rosmarinic acid interferes with the synthesis and degradation pathways of symbionts in *E. coli* and *S. aureus*, disrupts the tricarboxylic acid (TCA) cycle, and induces excessive ketone body accumulation, ultimately triggering bacterial apoptosis [71]. *P. frutescens* aldehyde induces necrosis and apoptosis in *Botrytis cinerea* and *Clostridium perfringens* spores. The underlying mechanisms involve MAPK signaling pathway disruption and compromised cell wall integrity in *B. cinerea*, autophagy induction through Ca^2+^- and ROS-mediated pathways, and interference with ribosomal function during transcription and translation, ultimately inhibiting biofilm formation in *Streptococcus mutans* [15,72,73].

Metabolomic analyses have demonstrated the antibacterial properties of *P. frutescens* against *Pseudomonas fluorescens* and elucidated its underlying mechanisms. *P. frutescens* induces substantial alterations in 128 metabolites, perturbing nucleotide metabolism, disrupting carbohydrate, amino acid, and lipid metabolism, and impairing cell wall biosynthesis, cell membrane integrity, and genetic material expression. These metabolic disruptions collectively suppress bacterial growth and ultimately result in cell death [74].

### 3.6. Lowers Blood Sugar

Recent pharmacological studies have underscored the hypoglycemic potential of *P. frutescens*. These effects are mainly attributed to bioactive compounds, including phenolic acids, flavonoids, terpenoids, and organic acids, which form the phytochemical basis of its antidiabetic activity [75]. Experimental studies suggest that *P. frutescens* oil confers dual therapeutic benefits in type 2 diabetic mouse models by mitigating insulin resistance and modulating gut microbiota through the PI3K/AKT signaling pathway [76].

Animal studies have shown that perillaldehyde, a key monoterpene component, attenuates oxidative stress in diabetic cardiomyopathy by inhibiting the PARP1-TRPM2-CaMKII/CaN pathway [77]. Furthermore, *P. frutescens* sprout extract (PFSE) exhibits significant metabolic regulatory effects through two primary mechanisms: suppression of gluconeogenic signaling pathways and activation of phosphorylated AMP-activated protein kinase (AMPK), thereby ameliorating symptoms in type 2 diabetic mice [78,79].

Luteolin supplementation (10 mg/kg/day for 4 weeks) in streptozotocin-induced diabetic mice demonstrated nephroprotective effects by suppressing the RIP140/NF-κB pathway while simultaneously enhancing insulin signaling in renal tissues [80]. Structural modification studies have yielded valuable insights into the bioactivity of polysaccharides. Sulfation markedly enhanced the α-glucosidase and α-amylase inhibitory activities of *P. frutescens* polysaccharide PLP-2-1, increasing inhibition rates from 71.77% to 76.06% and from 33.15% to 47.73%, respectively, resulting in the sulfated derivative S-PLP-2-1 [81].

### 3.7. Others

*P. frutescens* demonstrates a wide range of pharmacological activities, including antidepressant, anti-Alzheimer’s, sleep-promoting, anti-asthmatic, and fertility-enhancing properties. The functional mechanism is shown in Table 2. Luteolin-7-*O*-glucuronide (L7GN), a key bioactive flavonoid, alleviated stress-induced depression-like behaviors and adaptive responses in a murine sleep deprivation (SD) model [82]. The extracts of the plant show promising dermatological potential: rosmarinic acid, a principal phenolic component, promotes hair growth through time-dependent enhancement of cellular viability while inhibiting testosterone and dihydrotestosterone (DHT) activity, making it a strong candidate for the treatment and prevention of androgenetic alopecia (AGA) [83].

Perillaldehyde exerts neuroprotective effects in dopaminergic neurons, primarily by modulating G3BP-mediated stress granule (SG) formation, indicating its therapeutic potential for neurodegenerative diseases [84]. Furthermore, perillaldehyde possesses ideal physicochemical properties for transdermal drug delivery, acting as a novel permeation enhancer by enhancing lipid bilayer fluidity and reducing diffusion barriers in the stratum corneum [85]. Isoimperatorin accelerated cutaneous wound healing in dermatological repair by activating the mitogen-activated protein kinase/extracellular signal-regulated kinase (MAPK/ERK) pathway [86]. Agricultural applications were further demonstrated by *Streptomyces sp. NEAU-ZSY13*, an endophytic strain isolated from *P. frutescens* leaves, exhibiting broad-spectrum biocontrol activity against *Ralstonia solanacearum* (tomato bacterial wilt) and *Bipolaris sorokiniana* (wheat root rot), with control efficacy of 72.2% and 78.3%, respectively [87].

**Table 2:** Models and mechanisms of functions of *Perilla frutescens* extract.

Function	Cell/Animal Model	Mechanism of Action	References
Antioxidant	t-BHP-induced hepatotoxicity in rats	Modulates CYP1A1/2 activity and heme oxygenase-1 (HO-1) expression via nuclear factor erythroid 2-related factor 2 (NRF2) activation, mitigating oxidative liver damage.	Kang et al. [88]
Anti-inflammatory	COPD mouse model	Inhibits leukocytosis and neutrophilia in BALF; suppresses p38 MAPK/NF-κB p65 signaling, reducing inflammatory mediator production and neutrophil infiltration.	Wei et al. [89]
Antiviral	PK15 cells and PRV-infected mice	Luteolin inhibits viral replication by downregulating viral mRNA/gB protein expression, reduces apoptosis in PRV-infected cells, and enhances survival rates post-lethal challenge.	Men et al. [90]
Anticancer	Huh-7, Hep3B cells, and xenograft mice	Isoegomaketone suppresses hepatocellular carcinoma (HCC) growth via PI3K-Akt signaling pathway blockade.	Wang et al. [91]
Antimicrobial	*Trichophyton mentagrophytes*	Inhibits enolase expression, disrupting fungal glycolysis and energy metabolism, thereby suppressing growth.	Xu et al. [35]
Hypoglycemic	HFD/STZ-induced T2DM SD rats	Reduces hyperglycemia, ameliorates hepatic/intestinal tissue damage, and decreases glycogen accumulation via enhanced insulin signaling.	Wang et al. [75]
Anti-Alzheimer’s	5XFAD transgenic mice	Blocks Aβ aggregation, dissociates preformed Aβ fibrils, and prevents Aβ-induced LTP impairment and memory deficits.	Cho et al. [92]
Antidepressant	CUMS-induced depressed rats	Modulates monoaminergic neurotransmission and activates BDNF/TrkB signaling pathways.	Zhong et al. [93]
Sleep Promotion	Pentobarbital-induced sleep mice	Exhibits adenosine A1 receptor (A1R) agonism, enhances neuronal activity in sleep-promoting brain regions, and reduces activity in wakefulness-associated regions.	Joy et al. [94]
Anti-asthmatic	OVA-induced allergic asthma mice	Suppresses airway inflammation and immune dysregulation via inhibition of ERK, JNK, and p38 MAPK phosphorylation.	Cao et al. [95]
Hypolipidemic	Hyperlipidemic rats	Reduces serum lipid levels, inhibits lipid peroxidation, normalizes lipoprotein metabolism, and enhances antioxidant enzyme activity.	Feng et al. [96]
Fertility Enhancement	Human endometrial Ishikawa cells	Upregulates integrin β3/β5 expression via leukemia inhibitory factor (LIF)-dependent pathways, enhancing adhesion between endometrial and trophoblast cells.	Kim et al. [97]

## 4. Innovation in Food and Industrial Applications

### 4.1. Development of Functional Foods

*P. frutescens* oil is increasingly recognized as a multifunctional edible oil with broad industrial applications, owing to its natural composition and bioactive properties [11,98]. Its integration into margarine formulations underscores its versatility in lipid-based food products [99]. Nutritional analysis indicates that *P. frutescens* seed oil is particularly abundant in ω-3 polyunsaturated fatty acids [100], which mitigate intracellular lipid accumulation by markedly reducing serum levels of total cholesterol (TC), triglycerides (TG), low-density lipoprotein cholesterol (LDL-C), and high-density lipoprotein cholesterol (HDL-C) [101]. Spray-dried microencapsulation effectively stabilizes α-linolenic acid (ALA) in *P. frutescens* oil, enhancing its shelf life while maintaining its nutritional integrity. The anti-atherogenic properties of *P. frutescens* leaves have been evidenced by their ability to suppress lipid accumulation in arterial tissues [96]. Anti-obesity effects observed in 3T3-L1 adipocytes and rodent models highlight *P. frutescens* as a promising functional food for combating obesity and metabolic disorders [102]. Synergistic formulations, such as *P. frutescens*-chrysanthemum functional beverages, display enhanced antioxidant properties due to the synergistic interactions between polyphenols, flavonoids, and anthocyanins [103]. *P. frutescens* seed cake hydrolysate, particularly its rosmarinic acid-enriched fraction, significantly enhanced muscle mass and exercise endurance in mice, indicating its potential applications in sports nutrition and the development of meat substitutes [104]. The antioxidant, anticancer, anti-inflammatory, and antidiabetic properties of *P. frutescens* seed proteins (peptides) support their potential in the development of functional foods [105]. From an industrial perspective, the primary utilization of *P. frutescens* focuses on its extracts. These include standardized extracts, such as volatile oil-rich fractions for flavor enhancement, ethanolic extracts with antimicrobial properties suitable for food preservation, and other bioactive compound-rich extracts with applications in functional foods and nutraceuticals. The extracts or products derived from *Perilla frutescens* are suitable for large-scale production, comply with the legal and regulatory requirements for food products, and can maintain their quality over time (i.e., have a stable shelf life) when used in the food industry.

### 4.2. Natural Preservatives and Preservatives

PEO and its phenolic acid constituents demonstrate broad-spectrum antimicrobial activity. Among these, perillaldehyde demonstrates significant antibacterial activity, particularly against foodborne pathogens such as *E*. *coli*, positioning it as a promising natural preservative [106]. Perillaldehyde suppresses the growth of *Botrytis cinerea*, disrupts *Streptococcus* biofilm formation, and contributes to the preservation of seafood and fresh fruits [15,73]. A “sandwich” nanofiber membrane embedded with perillaldehyde extended the shelf life of refrigerated chicken by 6–10 days via sustained antimicrobial release [107].

PEO-loaded hydrogel beads effectively retain organic acids and total phenolics in strawberries, significantly reducing microbial counts and enhancing sensory attributes, particularly taste and color [108]. PEO microcapsules, fabricated with a core-to-shell ratio of 1.4:1, exhibit excellent encapsulation efficiency (91.5%) and a smoother surface morphology. These microcapsules help preserve high antioxidant and antibacterial activity during peach storage [109]. Nanoemulsified PEO prolonged the shelf life of frozen beef by 14 days by inhibiting lipid oxidation [110].

*P. frutescens* leaf extract effectively mitigates lipid and protein oxidation, reduces total volatile basic nitrogen (TVB-N) levels in surimi fish balls, and inhibits microbial growth in surimi-based products [111]. Composite edible films were developed by integrating PEO-glycerol monolaurate emulsions with chitosan and nisin. The formulated film exhibited an exceptional inhibition rate of 99.94% against *Staphylococcus aureus*, underscoring its potential as an antimicrobial packaging material for seafood preservation [112]. These studies provide valuable insights into the application of natural preservatives in the food industry and promote sustainable practices in food preservation.

### 4.3. Novel Food Additives

*P. frutescens* seed powder functions as a dual-purpose fat substitute in processed meats, reducing heterocyclic amine formation and mitigating protein oxidation in heat-treated chicken products [113]. Enzymatic hydrolysis of *P. frutescens* seed powder yields water-soluble dietary fiber with enhanced hydration properties, rendering it an ideal functional carbohydrate source for food additives and nutraceuticals [114]. In ruminant nutrition, a diet supplemented with 3% *P. frutescens* seed significantly enhanced growth performance and carcass quality in Tan lambs, while improving raw meat flavor profiles by increasing umami nucleotide content [115]. *P. frutescens* leaf supplementation activated pyrimidine metabolism pathways in rumen fluid and stimulated unsaturated fatty acid biosynthesis in milk, thereby enhancing flavor compound production [116].

The incorporation of PEO significantly enhanced the flavor profile of roasted Wuchang fish, increasing key volatile compounds such as α-terpineol, linalool, camphene, and β-pinene. This treatment markedly enhanced umami intensity, as evidenced by a guanosine monophosphate (GMP) content of 87.75 mg/g, which contributed to improved sensory attributes and textural properties [117]. Anthocyanins in *P. frutescens* leaves function as pH-responsive natural pigments, shifting between red, purple, and blue, thereby making them suitable for coloring beverages and confectioneries [118]. Co-pigmentation with rosmarinic acid stabilized anthocyanins and enhanced food coloration [119].

Fermentation of *P. frutescens* leaf extract (5%, 10%, 15%, and 20% *w*/*v*) using *Lactobacillus acidophilus* KCTC 3164 demonstrated that the melting rate, expansion ratio, DPPH radical scavenging activity, and total phenolic content in ice cream increased with higher amounts of *P. frutescens* leaf extract, whereas viscosity and pH showed an inverse correlation. Among the different concentrations, the 10% *P. frutescens* leaf fermented extract stood out as a novel additive for functional ice cream production [120]. Milk-based gummies were formulated using a 65:10 ratio of sweetened condensed milk and whey permeate, incorporating 18% *P. frutescens* syrup and 4% gelatin. These formulations exhibited high nutritional and sensory value, supporting their potential as novel functional food products [121].

## 5. Biology and Genetic Improvement

Advancements in genomics and metabolic engineering have positioned *P. frutescens* as a key candidate for genetic improvement and metabolic optimization. CRISPR-Cas9-mediated genome editing has been effectively employed to enhance agronomic traits, including biomass production and stress tolerance [122]. Key genes involved in leaf pigmentation, seed development, and trichome formation have been identified through comparative genomics, providing molecular tools for precision breeding [123,124].

The exceptionally high α-linolenic acid (ALA) content in *P. frutescens* seeds (up to 60.9% of total fatty acids) underscores the importance of elucidating its biosynthetic regulation, particularly for metabolic engineering applications [125]. Transcriptomic analysis has identified key enzyme transcripts associated with fatty acid and triacylglycerol (TAG) biosynthesis, laying a foundation for genetic and evolutionary research in *P. frutescens*, including inter-varietal comparisons [126]. Metabolomic analysis has further characterized distinct chemotypes in *P. frutescens*, categorizing them into monoterpenoid (MT) and phenylpropanoid (PP) types. The MT chemotypes include high phenolic acid (PA), perilla ketone (PK), high essential oil (EK), umbelliferone (PT), perilla alcohol (PL), shisofuran (SF), and pleurisy (C) types (Figure 2B,C), providing critical support for molecular marker-assisted breeding [127,128]. Moreover, multi-omics approaches have elucidated the diverse functions and potential applications of *P. frutescens*’s organs and tissues, providing theoretical insights for the targeted utilization of organ-specific metabolites [129].

## 6. Security and Standardization

### 6.1. Toxicity and Security

Comprehensive safety evaluations are essential before the application of *P. frutescens* derivatives in the food and pharmaceutical industries. The *Chinese-Pharmacopoeia-Commission 2020* stipulates inductively coupled plasma mass spectrometry (ICP-MS) for quantifying heavy metals in *P. frutescens* leaves, including cadmium (Cd), mercury (Hg), lead (Pb), arsenic (As), and copper (Cu) [130]. Pesticide residue analysis employs chromatographic methods to detect compounds such as hexachlorobenzene, quintozene, cis-chlordane, trans-chlordane, oxy-chlordane, heptachlor, heptachlor-exo-epoxide, and heptachlor-endo-epoxide. Perilla ketone exhibits a dual pharmacological profile in research, demonstrating anti-inflammatory and antimicrobial activities alongside potent pulmonary toxicity. Based on a presumed average body weight of 63 kg, the estimated safe intake of *P. frutescens* leaves is ≤5 g·d^−1^ to prevent perilla ketone-induced toxicity [131].

While both perilla alcohol and perillaldehyde hold Generally Recognized As Safe (GRAS) designation, perillaldehyde continues to face persistent scrutiny in scientific debates over its potential health risks. Perillaldehyde has an LC50 (the 50% lethal concentration) of 7.975 mg/L in zebrafish, while sublethal exposure at 4 mg/L induces morphological abnormalities and neurotoxicity [132]. Perillaldehyde demonstrates significant cytotoxicity in vitro and hepatotoxicity in vivo, necessitating precise dose regulation to mitigate these adverse effects. Despite its antimicrobial, anticancer, and anti-inflammatory properties, strict dosage control is imperative, as only low-to-moderate doses are deemed safe for human use to minimize risks of hepatotoxicity and neurodevelopmental disorders [133]. Furthermore, *P. frutescens* seeds present allergenic risks, with occupational asthma reported in individuals exposed to roasted seed fumes [134]. Oleosins have been identified as predominant allergenic proteins within the seed matrix [135].

Toxicological studies have determined a no-observed-adverse-effect level (NOAEL) of *P. frutescens* oil at 3 g/kg/day in beagle dogs; however, histopathological analyses indicate potential hepatolienal toxicity, necessitating further investigation [136]. Additionally, the risk of adulteration must be carefully monitored, as atmospheric solids analysis probe-mass spectrometry (ASAP-MS) can detect adulterants such as cinnamon oil at concentrations as low as 5% (*v*/*v*) with 93% accuracy [137].

### 6.2. Standardization

International regulations regarding pesticide residues in *P. frutescens* products vary considerably. The European Union (EU) implements a limit of detection-based standard, whereas China’s *P. frutescens* industry standard enforces a maximum residue limit of 0.05 mg/kg. Japan enforces a more stringent threshold of 0.01 mg/kg. Additionally, the regional standard of Inner Mongolia (DB15/T 3207-2024) mandates heavy metal limits for *P. frutescens* leaves, specifying maximum permissible levels of lead (≤5.0 mg/kg) and cadmium (≤0.3 mg/kg) [138]. Compliance with these standards is validated using atomic absorption spectroscopy, ensuring a recovery rate of ≥95%.

## 7. Conclusions

*P. frutescens*, a traditional medicinal and edible plant, has garnered significant research attention in recent years. It is abundant in essential nutrients and contains volatile oils, flavonoids, phenolic acids, and terpenes, which exhibit notable anti-inflammatory, anticancer, antioxidant, and antibacterial properties. Furthermore, *P. frutescens* exhibits significant potential in functional foods and natural food additives, particularly as a source for developing natural pigments, flavoring agents, and preservatives. Notably, *P. frutescens* oil, distinguished by its high omega-3 fatty acid content, is recognized as a valuable functional food ingredient. This review systematically examines the chemical composition and bioactive properties of *P. frutescens*, outlining a comprehensive research framework that encompasses resource utilization, mechanistic insights, technological advancements, and regulatory considerations, thereby supporting its high-value applications in functional foods and sustainable industries.

Although the pharmacological properties of *P. frutescens* bioactive compounds have been extensively investigated, their safety evaluations remain largely confined to preclinical studies, with clinical research still lacking. Additionally, the structural diversity and complexity of *P. frutescens* bioactive constituents, particularly polysaccharides, pose significant challenges for in-depth investigation. While the primary bioactive components of *P. frutescens* have been largely elucidated, their structural characteristics, pharmacological mechanisms, and metabolic pathways require further investigation. Future research should prioritize comprehensive safety evaluations and clinical studies of *P. frutescens* bioactive compounds, while utilizing advanced analytical techniques to elucidate their intricate chemical structures. These efforts will facilitate the high-value utilization of *P. frutescens* in pharmaceuticals, functional foods, and sustainable industrial applications.

## Figures and Tables

**Table 1:** Statistical analysis of research directions on *Perilla frutescens* based on the Web of Science database (2021–2025). Specific data can be found in Supplementary Table S1.

Year	Total Publications	Primary Research Areas				
		Chemical Composition Research	Pharmacological Activity Research	Food Industry Applications	Genetic Improvement Research	Other Studies (Safety Standards/Heavy Metals/Stresses/Environment/ Quality/Pests, and Diseases/Clinical)
2025	16	5	4	1	3	3
2024	143	19	44	16	19	45
2023	154	26	47	24	20	37
2022	148	29	38	25	17	39
2021	120	28	38	10	17	27

## Data Availability

No new data were created or analyzed in this study. Data sharing is not applicable to this article.

## Supplementary Materials

The following supporting information can be downloaded at: https://www.mdpi.com/article/10.3390/foods14071252/s1, Table S1: Publications on *Perilla frutescens* from 2021 to 2025.
